# Ocrelizumab dose selection for treatment of pediatric relapsing–remitting multiple sclerosis: results of the OPERETTA I study

**DOI:** 10.1007/s00415-024-12879-z

**Published:** 2025-01-15

**Authors:** Soe Mar, Massimiliano Valeriani, Barbara Steinborn, Teri Schreiner, Emmanuelle Waubant, Massimo Filippi, Katarzyna Kotulska, Maria Mazurkiewicz-Beldzinska, Bouchra El Azzouzi, Chien-Ju Lin, Yun-An Shen, Heidemarie Kletzl, Joanna Evershed, Alexandra Hogea, Corinne Manlius, Ulrike Bonati, Brenda Banwell

**Affiliations:** 1 Pediatric Multiple Sclerosis and Demyelinating Diseases Center, Washington University in St. Louis, St. Louis, MO USA; 2 Developmental Neurology Unit, Bambino Gesù Children Hospital, Rome, Italy; 3 Systems Medicine Department, University of Tor Vergata, Rome, Italy; 4 Department of Developmental Neurology, Poznań University of Medical Sciences, Poznań, Poland; 5 Departments of Neurology and Pediatrics, University of Colorado, Aurora, CO USA; 6 Department of Neurology, University of California San Francisco, San Francisco, CA USA; 7 Neurology Unit, IRCCS San Raffaele Scientific Institute, Milan, Italy; 8 Vita-Salute San Raffaele University, Milan, Italy; 9 Department of Neurology and Epileptology, The Children’s Memorial Health Institute, Warsaw, Poland; 10 Department of Developmental Neurology, Chair of Neurology, Medical University of Gdańsk, Gdańsk, Poland; 11 F. Hoffmann-La Roche Ltd, Basel, Switzerland; 12 Roche Products Ltd, Welwyn Garden City, UK; 13 Genentech, Inc., South San Francisco, CA USA; 14 Division of Child Neurology, Children’s Hospital of Philadelphia, Departments of Neurology and Pediatrics, Perelman School of Medicine, University of Pennsylvania, Philadelphia, PA USA

**Keywords:** Pediatric-onset multiple sclerosis, Pediatric, Dose-finding, Ocrelizumab

## Abstract

**Background:**

The presented study identified the appropriate ocrelizumab dosing regimen for patients with pediatric-onset multiple sclerosis (POMS).

**Methods:**

Patients with POMS aged 10–17 years were enrolled into cohort 1 (body weight [BW] < 40 kg, ocrelizumab 300 mg) and cohort 2 (BW ≥ 40 kg, ocrelizumab 600 mg) during a 24-week dose-exploration period (DEP), followed by an optional ocrelizumab (given every 24 weeks) extension period. Primary endpoints: pharmacokinetics, pharmacodynamics (CD19^+^ B-cell count); secondary endpoint: safety; exploratory endpoints: MRI activity, protocol-defined relapses, Expanded Disability Status Scale (EDSS) score change.

**Results:**

A total of 23 patients (cohort 1: n = 6, age 10–12 years, BW 27.0–39.0 kg; cohort 2: n = 17, age 11–17 years, BW 42.1–108.4 kg) were enrolled. Median treatment duration was 120 (range, 24–193) weeks at the primary analysis cutoff (October 5, 2023). Overall, the pharmacokinetic data were within the range observed at 600 mg in adult patients with MS; however, the exposure at 300 mg in patients < 40 kg was lower than with 600 mg in patients ≥ 40 kg. Shifting the cutoff to 35 kg would provide better exposure to patients with 35–40 kg body weight. Sustained, near-complete B-cell depletion was observed. The safety profile was consistent with that in adults. EDSS scores remained stable; no clinical relapses were observed.

**Conclusion:**

A dosing regimen of 300 mg ocrelizumab for patients < 35 kg, and 600 mg for patients ≥ 35 kg (every 24 weeks), was selected for the phase 3 OPERETTA II trial (NCT05123703).

**Trial registration:**

ClinicalTrials.gov: NCT04075266.

**Supplementary Information:**

The online version contains supplementary material available at 10.1007/s00415-024-12879-z.

## Introduction

Multiple sclerosis (MS) is a chronic, immune-driven, neuroinflammatory, and neurodegenerative disease, characterized by the gradual accumulation of damage to the central nervous system [1]. Pediatric-onset MS (POMS) is rare, with only 2–5% of patients with MS experiencing initial disease symptoms before the age of 18 years [2, 3]. Of these, over 95% follow a relapsing–remitting course [4]. Relapsing–remitting POMS is a highly active disease, with frequent relapses and rapid accrual of MRI lesions [3, 4]. Although patients with POMS show a greater ability to compensate and recover after disease flares and have slower progression to disability, they reach disability milestones at a younger age than those with adult-onset MS (AOMS) [5]. Despite this, treatment options remain limited, and, currently, in Europe, only three disease-modifying therapies (DMTs) are approved for patients with POMS aged below 18 years; these approvals vary in other countries and territories [6]. Thus, the lack of approved therapies leads to off-label use of DMTs, especially of high-efficacy DMTs, highlighting the critical need for clinical trials in POMS [7].

Ocrelizumab (OCR; Ocrevus^®^, Genentech, Inc., South San Francisco, CA, USA), is a recombinant, humanized monoclonal antibody that selectively depletes B cells expressing the cluster of differentiation (CD)20 marker [8, 9] while preserving the capacity for B-cell reconstitution and pre-existing humoral immunity. OCR is a high-efficacy DMT approved for the treatment of adults with relapsing and primary progressive MS [10, 11], with over 10 years of safety and efficacy experience in clinical trials and that has been used to treat over 350,000 adult patients in both clinical trial and post-marketing environments [12–14].

The objective of the OPERETTA I study was to identify a dosing regimen for patients with POMS that achieved a comparable pharmacokinetic (PK), pharmacodynamic (PD), and safety profile to the approved 600 mg intravenous (IV) dose for adult patients with MS. The selected dosing regimen will be further assessed for safety and efficacy in the phase 3 OPERETTA II study.

## Methods

### Trial design and procedures

**Fig. 1:**
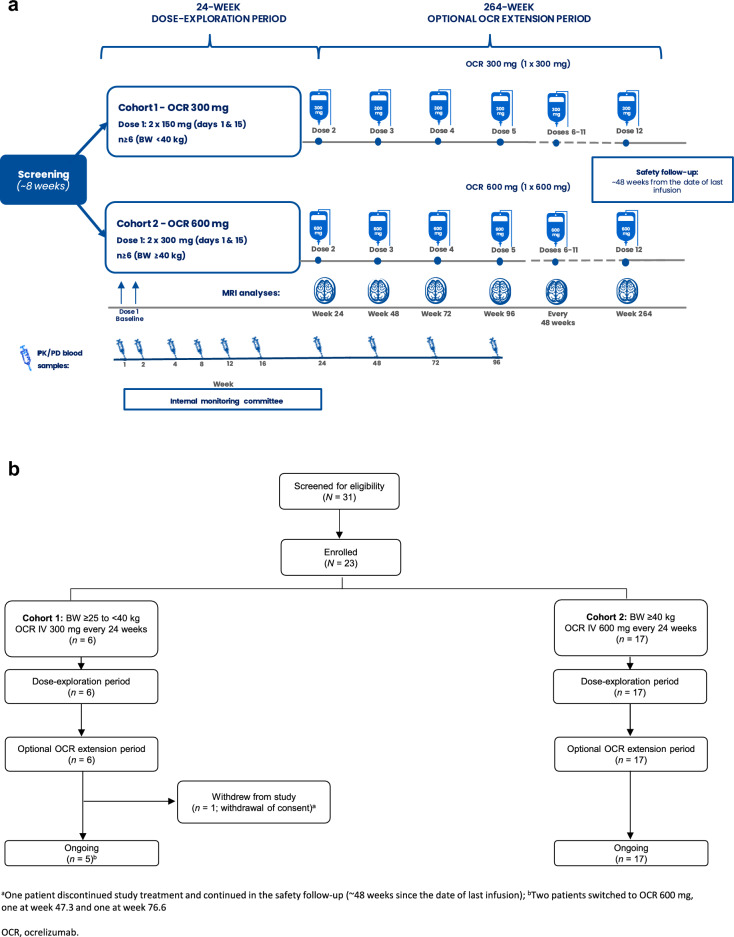
OPERETTA I **A** study design and **B** patient disposition. ^a^One patient discontinued study treatment and continued in the safety follow-up (~ 48 weeks since the date of last infusion). ^b^Two patients switched to OCR 600 mg, one at Week 47.3 and one at Week 76.6. BW, body weight; OCR, ocrelizumab; PD, pharmacodynamic; PK, pharmacokinetic

OPERETTA I (NCT04075266) is an open-label, parallel-group, phase 2 study investigating the PK, PD, safety, and tolerability of OCR in children and adolescents with relapsing–remitting MS (RRMS) aged 10–17 years. The study consisted of four phases: screening (up to 8 weeks); a dose-exploration period (DEP; 24 weeks); an optional OCR extension period (OOE; 264 weeks); and safety follow-up (minimum 48 weeks; Fig. 1A).

Eligible patients were assigned to one of two cohorts based on their body weight (BW) at the time of enrollment. Patients with a BW ≥ 25 kg and < 40 kg were assigned to cohort 1 to receive 300 mg OCR IV every 24 weeks; patients with a BW ≥ 40 kg were assigned to cohort 2 to receive 600 mg OCR IV every 24 weeks. As per the adult prescribing information, the first dose of OCR was administered as two IV infusions of half the dose 14 days apart (i.e. 2 × 150 mg in cohort 1 and 2 × 300 mg in cohort 2). Subsequent IV doses were administered as single infusions every 24 weeks. Patients in cohort 1 who reached a stable BW of ≥ 40 kg during the study were switched to the 600 mg dosing regimen (with the first dose again administered as two IV infusions of 300 mg 14 days apart).

Two weeks before each infusion of OCR, a visit was required to collect blood samples and patient information. This enabled the study investigator to confirm that the patient met the retreatment criteria (see Supplementary Information for further information) at the time of infusion. All patients received mandatory prophylactic treatment with an antihistamine (e.g. diphenhydramine) and methylprednisolone dose adjusted to weight (patients < 40 kg received 2 mg/kg and patients ≥ 40 kg received 100 mg prior to the infusion).

Patients underwent regular safety monitoring during the study, including assessment of the nature, frequency, and severity of adverse events (AEs).

### Patients

The trial enrolled patients aged 10–17 years with a BW ≥ 25 kg, a diagnosis of RRMS (per the International Pediatric Multiple Sclerosis Study Group criteria for pediatric MS Version 2012 [15] and/or 2017 revised McDonald criteria [16]) and an Expanded Disability Status Scale (EDSS) score of 0–5.5 at screening. Patients were also required to: be DMT treatment naive (or have received < 6 months of a DMT [e.g. interferons or glatiramer acetate] within the past year) and have had ≥ 2 relapses in the last 2 years (with ≥ 1 relapse occurring in the previous year), ≥ 2 relapses in the last 2 years with ≥ 1 gadolinium (Gd)-enhancing lesion on T1-weighted brain MRI at any time within the previous year, or ≥ 1 relapse in the previous year and ≥ 1 Gd-enhancing lesion on T1-weighted brain MRI at any time within the previous year. Patients who had received ≥ 6 months of continuous DMT treatment within the past 1 year were required to have had ≥ 1 relapse or have ≥ 1 Gd-enhancing lesion on T1-weighted brain MRI. Key exclusion criteria included known presence or suspicion of other neurologic disorders that may mimic MS, positivity for aquaporin 4 immunoglobulin G, or a diagnosis of myelin oligodendrocyte glycoprotein antibody–associated disease (see Supplementary Information for further inclusion criteria)*.*

### Study objectives and endpoints

#### Primary pharmacokinetic and pharmacodynamic objectives

The primary PK objective was to characterize the OCR PK profile in children and adolescents based on measuring the OCR serum concentration over time (at predefined time points). The primary PD objective was to evaluate the relationship between drug exposure and PD in children and adolescents based on levels of CD19^+^ B cells in the blood. The absolute CD19^+^ B-cell count in blood was measured at a central laboratory using the BD Multitest™ 6-Color TBNK reagents and BD Trucount tubes on a FACSCanto II cytometer (BD Biosciences, San Jose, CA, USA).

#### Safety objective

The safety and tolerability of OCR were evaluated based on the following endpoints: occurrence and severity of AEs; changes from baseline in vital signs, electrocardiograms, and clinical laboratory results; and locally reviewed MRIs (for non-MS central nervous system pathology). The severity of AEs was graded according to the National Cancer Institute Common Terminology Criteria for Adverse Events (NCI CTCAE) version 5.0.

#### Immunogenicity objective

Immunogenicity was assessed via the presence of antidrug antibodies (ADAs) to OCR, based on the incidence of treatment-emergent ADAs, during the DEP and OOE relative to baseline.

#### Exploratory objectives

Exploratory parameters were: protocol-defined relapses (accompanied by objective neurologic worsening consistent with an increase of at least 0.5 points in EDSS score; an increase of 2 points in one of the appropriate Functional System Score [FSS] scales; or an increase of 1 point in at least two of the appropriate FSS scales [i.e. pyramidal, ambulation, cerebellar, brainstem, sensory, or visual]), as compared with the most recent evaluation of the EDSS score; change in EDSS score from the most recent evaluation; number of patients with no evidence of disease activity (NEDA); change in total number of T1 Gd-enhancing lesions at Week 12; change in total number of new and/or enlarging (N/E) T2 hyperintense lesions followed up throughout the trial at 24-week intervals; and change in Symbol Digit Modalities Test (SDMT) score, administered using the oral version, to assess cognitive function throughout the trial at 24-week intervals.

### Data analysis

The sample size was chosen based on practical considerations and feasibility.

Cohort characteristics were summarized using means, medians, and ranges for continuous variables and frequencies (%) for categorical variables. All patients received study treatment and were included in the analyses.

Blood was drawn for PK analysis during the DEP (Fig. 1). OCR serum concentration was measured with an ELISA using established and validated immunoassay procedures. The concentration data were analyzed using the population PK method via non-linear mixed-effects modeling.

### Standard protocol approvals, registrations, and patient consent

The trial protocol (ClinicalTrials.gov identifier: NCT04075266) was approved by the relevant institutional review boards/ethics committees. The most recent signed copy of the Protocol is available as supplementary material. All patients (or the patients’ legally authorized representatives) provided written informed consent.

### Data availability

For up-to-date details on Roche’s global policy on the sharing of clinical information and how to request access to related clinical study documents, see https://go.roche.com/data_sharing. Anonymized records for individual patients across more than one data source external to Roche cannot, and should not, be linked, due to a potential increase in risk of patient re-identification. For eligible studies, qualified researchers may request access to individual patient-level clinical data through a data request platform. At the time of writing, this request platform is Vivli (https://vivli.org/ourmember/roche/).

## Results

### Patient demographics, disease characteristics, and disposition

Twenty-three patients were enrolled between January 9, 2020, and April 20, 2023, across 12 centers in three participating countries (Italy, Poland, and the USA) (Fig. 1B). Six patients were enrolled into cohort 1 (BW < 40 kg; OCR 300 mg) and 17 patients into cohort 2 (BW ≥ 40 kg; OCR 600 mg). All 23 enrolled patients completed the 24-week DEP and continued in the OOE. One patient in cohort 1 withdrew from the study during the OOE (after Dose 4) due to patient withdrawal of consent/assent. Patient demographics and baseline disease characteristics are presented in Table 1. Median age at screening was 11.2 (range, 10–12) years in cohort 1 and 15.3 (range, 11–17) years in cohort 2. Mean weight at screening was 35.3 (range, 27–39) kg in cohort 1 and 63.6 (range, 42.1–108.0) kg in cohort 2. Median duration since onset of MS symptoms was 0.3 (range, 0.3–0.5) years in cohort 1 and 0.4 (range, 0.1–7.1) years in cohort 2. Four patients in cohort 2 had previously received DMTs (dimethyl fumarate [n = 3], fingolimod [n = 1], and glatiramer acetate [n = 1]). Median EDSS score at screening was 0.8 (range, 0–2.5) in cohort 1 and 1.0 (range, 0–3.5) in cohort 2.

**Table 1:** Patient demographic and clinical characteristics at baseline

Variable	OCR 300 mg (n = 6)	OCR 600 mg (n = 17)
Age at screening, years, mean (range)	11.2 (10–12)	15.3 (11–17)
Sex, n (%)		
Female	5 (83.3)	10 (58.8)
Male	1 (16.7)	7 (41.2)
Race, n (%)		
White	5 (83.3)	16 (94.1)
Black	–	1 (5.9)
Asian	1 (16.7)	–
Weight at screening, kg, mean (range)	35.3 (27.0–39.0)	63.6 (42.1–108.4)
Tanner stage ≥ 2, n (%)	2 (100)	9 (100)
Previous DMT use, n	0	4
EDSS score, median (range)	0.8 (0.0–2.5)	1.0 (0.0–3.5)
Duration since MS symptom onset, years, median (range)	0.3 (0.3–0.5)	0.4 (0.1–7.1)
Number of relapses in prior year, mean (SD)	1.2 (0.41)	0.9 (0.24)
Number of T1 Gd+ lesions, mean (SD)	2.8 (4.12)	4.4 (11.91)
Number of hyperintense T2 lesions, mean (SD)	40.00 (16.92)	49.41 (48.86)

DMT, disease-modifying therapy; EDSS, Expanded Disability Status Scale; Gd+ , gadolinium-enhancing; MS, multiple sclerosis; OCR, ocrelizumab; SD, standard deviation

This manuscript presents the primary analysis results of the OPERETTA I study, at the completion of the 24-week DEP, and cumulative data up to a clinical cutoff date (CCOD) of October 5, 2023.

### Exposure to ocrelizumab

At the CCOD, including the DEP and OOE, for cohorts 1 and 2, respectively, the median treatment duration was 84.6 (range, 24–148) weeks and 120.3 (range, 95–193) weeks and the median number of doses received was 4.5 (range, 2–8) and 6.0 (range, 5–9). Of note, as of the CCOD, two of the six patients in cohort 1 had switched to the higher OCR dose of 600 mg; the switches occurred at 47.3 and 76.6 weeks.

### Pharmacokinetic analysis

PK data were obtained from six patients receiving 300 mg OCR in cohort 1 (< 40 kg) and 17 patients receiving 600 mg OCR in cohort 2 (≥ 40 kg). The 300 mg and 600 mg doses were selected based on predictions for the respective BW categories, as BW is the most relevant covariate for the PK of OCR.

The OCR IV population PK model in adult patients with MS described the observed PK data in POMS from this study well. Based on the estimated PK parameters, the concentration–time course was simulated, and individual areas under the concentration–time curves for the 24-week dosing interval and peak concentrations were predicted (Table 2). The observed exposure was overall within the exposure range in adult patients with MS. However, the exposure in the < 40 kg group after 300 mg was on average lower than with 600 mg in the ≥ 40 kg group. Two potential dosing regimens were subsequently simulated: the first was the dosing regimen applied in this study, with 300 mg OCR IV for patients with a BW < 40 kg and 600 mg for patients with a BW ≥ 40 kg; the second option was a dosing regimen of 300 mg for patients with a BW < 35 kg and 600 mg for patients with a BW ≥ 35 kg. Using 35 kg as the cutoff resulted in exposure values for pediatric patients weighing 35–40 kg that were closer to the upper end of the adult exposure range. In contrast, a cutoff of 40 kg was predicted to result in exposure values closer to the lower end of the adult exposure range. As it was considered important to ensure adequate drug exposure to enable optimal treatment benefit in patients with POMS from the start of treatment, particularly in younger patients with early disease onset, it was decided to shift the BW cutoff to 35 kg. For patients < 35 kg BW, a dosing regimen of 300 mg is considered appropriate, as 600 mg would bring these patients above the exposure range established in adult patients with MS at the approved dose of 600 mg IV. A dosing regimen of 300 mg OCR IV for patients with a BW < 35 kg and 600 mg OCR IV for patients with a BW ≥ 35 kg, administered every 24 weeks, was selected to be assessed further in the phase 3 efficacy and safety study in POMS.

**Table 2:** Median (range) of predicted AUC_w1–24_ during DEP (µg/mL∙day)

	BW					
	< 35 kg	< 40 kg	≥ 40 kg	40–60 kg	60–80 kg	≥ 80 kg
Pediatric RRMS (2 × 150 mg if BW < 40 kg; 2 × 300 mg if BW ≥ 40 kg)	n = 2; 2583 (2077–3090)	n = 6; 2187 (1565–3090)	n = 17; 3197 (1781–4339)	n = 8; 3322 (3060–4339)	n = 6; 3348 (2748–3730)	n = 3; 1983 (1781–2110)
PPMS (2 × 300 mg)	–	–	n = 482; 2836 (369–6430)	n = 124; 3564 (1827–6430)	n = 209; 2873 (369–4494)	n = 149; 2230 (720–4796)
RMS (2 × 300 mg)	–	n = 1; 3520	n = 781; 2835 (1261–8849)	n = 155; 3602 (1858–8849)	n = 343; 2977 (1413–6889)	n = 283; 2332 (1261–3655)

AUC_w1–24_, area under the concentration–time curve for the 24-week dosing interval; BW, body weight; DEP, dose-exploration period; PPMS, primary progressive multiple sclerosis; RMS, relapsing multiple sclerosis; RRMS, relapsing–remitting multiple sclerosis

### Pharmacodynamic analysis

**Fig. 2:**
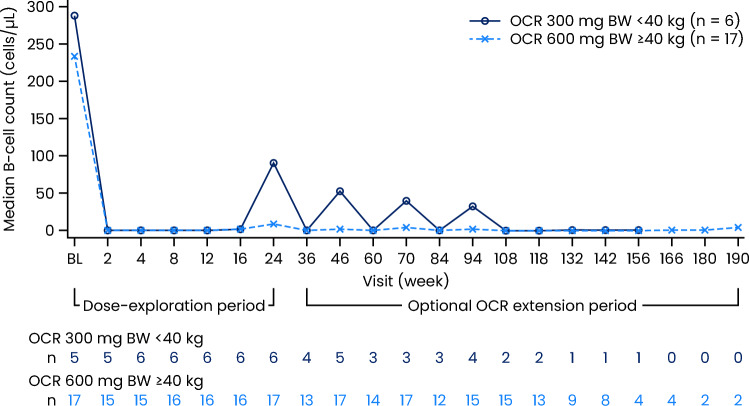
Median CD19^+^ B-cell count over time (scheduled visits only; safety-evaluable patients). BL, baseline; CD, cluster of differentiation; OCR, ocrelizumab

OCR treatment led to rapid and near-complete CD19^+^ B-cell depletion in blood, which was sustained up to the CCOD. The baseline B-cell value was the last B-cell count assessment prior to receiving the first dose of study drug, while the first post-dose value was assessed at Week 2. Patients in cohort 1 exhibited a substantial decrease in B-cell count from baseline to Week 2, with the median cell count dropping from 287.0 cells/µL to 2.0 cells/µL (Fig. 2). Similarly, B-cell depletion was achieved in patients in cohort 2, with the median cell count reducing from 234.0 cells/µL to 0.0 cells/µL at Week 2. At Weeks 4, 8, 12, and 16, median B-cell count remained low, and no B-cell repletion (defined as having a CD19^+^ B-cell count return to baseline value or age-appropriate lower limit of normal [LLN], whichever was lower) was observed [17, 18]. At Week 24, prior to receiving the second dose of OCR, some B-cell reconstitution was observed in both cohorts. While in the majority of patients the B-cell reconstitution level remained lower than the repletion threshold, one patient from each cohort (cohort 1: 1/6 patients; cohort 2: 1/17 patients) had their B-cell count return to above the LLN. At Week 24, the median B-cell count was 91.0 cells/µL in cohort 1 and 9.0 cells/µL in cohort 2. During the OOE period, repletion events were only observed in one patient in cohort 1 at Week 46 and Week 94 (Supplementary table S1).

### Safety, tolerability, and immunogenicity

#### Adverse events

The incidence of AEs among all patients receiving OCR in cohort 1 and 2 is summarized in Table 3. Overall, no new safety signals were identified. In cohort 1, a total of five patients (83.3%) reported AEs and only one patient (16.7%) experienced a grade 3 AE as the worst grade. Of patients with ≥ 1 AE, one patient had all AEs resolved and four patients had ≥ 1 unresolved. In cohort 2, all patients (100%) reported ≥ 1 AE with the worst grades being 3 and 4 in 35.3%, and 5.9% of patients, respectively. Six patients (35.3%) had their AEs resolved and 11/17 (64.7%) patients had ≥ 1 unresolved.

**Table 3:** Frequency and severity of AEs and timing and symptoms of IRRs

	OCR 300 mg (n = 6)	OCR 600 mg (n = 17)
Patients with ≥ 1 AE, n (%)	5 (83.3)	17 (100.0)
Total AEs, n	81	320
AEs by severity (worst grade), n of patients (%)		
Grade 1	0 (0.0)	1 (5.9)
Grade 2	4 (66.7)	9 (52.9)
Grade 3	1 (16.7)	6 (35.3)
Grade 4	0 (0.0)	1 (5.9)
Patients receiving treatment for AEs, n (%)	5 (83.3)	17 (100.0)
Patients with ≥ 1 unresolved or ongoing non-fatal AE, n (%)	4 (66.7)	11 (64.7)
Patients with ≥ 1 SAE, n (%)	1 (16.7)	4 (23.5)
Patients with ≥ 1 AE deemed related to study drug by PI, n (%)	5 (83.3)	14 (82.4)
Patients with AE leading to treatment discontinuation (including IRRs), n	0	0
Most frequent AEs, n of events (n of patients)		
IRRs	5 (3)	45 (14)
Headache	12 (2)	29 (8)
COVID-19	3 (3)	11 (11)
Abdominal pain	2 (2)	3 (2)
Abdominal pain upper	1 (1)	–
Pyrexia	–	7 (6)
Upper respiratory tract infection	–	8 (6)
Patients with ≥ 1 IRR, n (%)	3 (50.0)	14 (82.4)
Total number of IRRs at each dose, n		
Dose 1	3	14
Dose 2	1	6
Dose 3	0	6
Dose 4	0	4
Dose 5	1	5
Dose 6	0	5
Dose 7	0	2
Dose 8	0	2
Dose 9	0	0
Most frequent symptoms of IRRs, n of events (n of patients)		
Headache	1 (1)	20 (7)
Throat irritation	–	14 (4)
Fatigue	–	6 (5)
Rash	–	6 (3)
Myalgia	–	5 (2)
Dizziness	1 (1)	–
Flushing	1 (1)	–
Pallor	1 (1)	–
Nasal dryness	2 (1)	–
Dyspnea	1 (1)	–
Hot flush	–	4 (1)

AE, adverse event; COVID-19, coronavirus disease 2019; IRR, infusion-related reaction; OCR, ocrelizumab; PI, principal investigator; SAE, serious adverse event

Of safety events of interest, 49 infusion-related reactions (IRRs) were observed in 17 patients, primarily occurring at the first dose. In cohort 2, fourteen patients (14/17; 82.4%) experienced a total of 45 IRRs during the study. Headache was the most frequently reported IRR symptom (7 patients, 41.2%) followed by throat irritation (4 patients; 23.5%). In both cohorts, all IRRs events were reported as non-serious and the majority had the most extreme grade of 1 or 2. In cohort 2, one patient (7.1%) experienced a non-serious IRR with a most extreme intensity of grade 3 during the first infusion. Symptoms associated with this grade 3 IRR included dyspnea, rhinorrhea, and urticaria; all these events were reported as resolved. All IRR events in both study cohorts were assessed as related to the study drug and all events were reported as resolved. No IRR events led to dose delay or treatment discontinuation.

The most frequent infections reported were COVID-19 infections in 14 patients (60.9%), nasopharyngitis in seven patients (30.4%) and upper respiratory tract infection in seven patients (30.4%). Most reported infections were non-serious, with a most extreme intensity of grade 2. In cohort 1, one serious adverse event (SAE; appendicitis) was reported in one patient. In cohort 2, SAEs were reported in four patients (23.5%) and all were grade 3 except for one patient who reported grade 4 increased blood creatine phosphokinase and alanine transaminase increase, which started before study treatment initiation, with no underlying cause despite intensive workup. All SAE events were resolved without sequelae.

There were no pregnancies, and no treatment-related complications or abnormalities were reported. Overall, the safety profile was comparable to the one observed in adult patients treated with 600 mg OCR [19].

### Immunogenicity

From baseline up to the CCOD, no treatment-emergent ADAs to OCR were detected.

### Clinical exploratory endpoints

Following initiation of treatment with OCR, no clinical relapses were reported in any patients in either cohort.

The median change in EDSS score from baseline to Week 24 was − 0.25 (range, − 1.5, 0) in cohort 1 and 0 (range, − 1.5, 1) in cohort 2. At Week 48, four (80.0%; 4/5) and 14 (82.4%; 14/17) patients had improved or stable EDSS scores (defined as an EDSS score at the clinical visit being less than or equal to the EDSS score at baseline) in cohorts 1 and 2, respectively; at Week 96, the corresponding numbers of patients were three (100.0%; 3/3) and 14 (82.4%; 14/17) (Table 4).

**Table 4:** Proportion of patients with NEDA, and mean SDMT scores in all epochs

	Week 48		Week 96	
n (%)	OCR 300 mg^a^ (n = 6)	OCR 600 mg (n = 17)	OCR 300 mg^b^ (n = 6)	OCR 600 mg (n = 17)
NEDA				
Patients with relapse	0 (0.0)	0 (0.0)	0 (0.0)	0 (0.0)
Patients with improved or stable EDSS^c^	4 (80.0)	14 (82.4)	3 (100.0)	14 (82.4)
Patients with N/E T2 lesions	0 (0.0)	1 (5.9)	0 (0.0)	0 (0.0)
Patients with NEDA^d^	4 (80.0)	13 (76.5)	3 (100.0)	14 (82.4)
Patients with change in SDMT score ≥ 4 points	3 (60.0)	10 (62.5)	3 (100.0)	12 (75.0)
Patients with change in SDMT score ≥ 8 points	1 (20.0)	9 (56.3)	3 (100.0)	10 (62.5)

	OCR 300 mg (n = 6)		OCR 600 mg (n = 17)		Total (N = 23)	
	Mean (SD) value at visit	Change from baseline	Mean (SD) value at visit	Change from baseline	Mean (SD) value at visit	Change from baseline
SDMT						
Baseline	n = 6; 43.00 (7.87)	–	n = 16; 54.56 (21.97)	–	n = 22; 51.41 (19.68)	–
Week 24	n = 6; 47.83 (7.76)	4.83 (8.06)	n = 17; 61.53 (20.56)	7.50 (7.60)	n = 23; 57.96 (18.94)	6.77 (7.63)
Week 48	n = 5; 46.60 (8.65)	4.20 (4.60)	n = 17; 61.53 (21.21)	7.56 (7.56)	n = 22; 58.14 (19.95)	6.76 (7.02)
Week 72	n = 3; 52.67 (9.07)	7.33 (7.23)	n = 16; 62.06 (20.58)	11.07 (8.95)	n = 19; 60.58 (19.35)	10.44 (8.61)
Week 96	n = 3; 57.00 (7.00)	10.33 (3.21)	n = 17; 65.65 (22.02)	11.25 (9.52)	n = 20; 64.35 (20.58)	11.11 (8.76)

EDSS, Expanded Disability Status Scale; N/E, new and/or enlarging; NEDA, no evidence of disease activity; OCR, ocrelizumab; SD, standard deviation; SDMT, Symbol Digit Modalities Test

^a^Five patients in the 300 mg cohort completed the Week 48 visit; therefore, the percentages are based on n = 5

^b^Three patients in the 300 mg cohort completed the Week 96 visit; therefore, the percentages are based on n = 3

^c^Stable EDSS is defined as an EDSS score at the clinical visit of less than or equal to the EDSS score at baseline

^d^NEDA status was defined as the combination of absence of protocol-defined relapses with stable or improved EDSS score and absence of N/E T2 lesions

In cohort 1 (n = 6), a total of 17 T1 Gd-enhancing lesions were observed in six patients at baseline. In cohort 2 (n = 17), a total of 75 T1 Gd-enhancing lesions were observed in 17 patients at baseline (Table 5); this number is driven by one patient who had 49 T1 Gd-enhancing lesions at baseline. No T1 Gd-enhancing lesions were observed at Week 12 in any patient in either cohort. In cohort 1, the mean number of hyperintense T2 lesions at baseline was 40.0 (standard deviation [SD], 16.9; range, 9–55), with four patients showing 24 (range, 2–15) N/E T2 lesions at Week 12, and no N/E lesions from Week 24 onward, up to the CCOD for the patients with available data (Table 5). In cohort 2, the mean number of hyperintense T2 lesions at baseline was 49.4 (SD, 48.9; range, 13–225). The patient with 225 hyperintense T2 lesions at baseline also had a high baseline number of T1 Gd-enhancing lesions, with 49 lesions observed. In cohort 2, at Week 12, 11 patients showed N/E T2 lesions with a total number of 65 (range, 1–34), with the number reducing to three patients showing three N/E T2 lesions at Week 24 and only one N/E T2 lesion observed in one patient at Week 48. No new lesions were observed at Weeks 72 or 96 among the 17 patients.

**Table 5:** Number of T1 Gd+ and N/E T2 lesions at specified time points

	OCR 300 mg (n = 6)	OCR 600 mg (n = 17)
T1 Gd+ lesions, n (n of patients completing the visit)		
Baseline	17 (6)	75 (17)
Week 12	0 (6)	0 (17)
T2 lesions at baseline, mean (SD) [range]	40.0 (16.9) [9–55]	49.4 (48.9) [13–225]
N/E T2 lesions, n (n of patients completing the visit)		
Week 12	24 (6)	65 (17)
Week 24	0 (6)	3 (17)
Week 48	0 (5)	1 (17)
Week 72	0 (3)	0 (17)
Week 96	0 (2)	0 (17)

Gd+ , gadolinium-enhancing; N/E, new and/or enlarging; OCR, ocrelizumab; SD, standard deviation

NEDA status, defined as the combination of absence of protocol-defined relapses with stable or improved EDSS score and absence of N/E T2 lesions, is provided in Table 4. Overall, 17/22 (77.2%) patients met NEDA status at Week 48 and 17/20 (85.0%) patients met NEDA status at Week 96.

The mean SDMT score at baseline was 43.0 (SD, 7.9) in cohort 1 and 54.6 (SD, 22.0) in cohort 2. The mean change in SDMT score from baseline to Week 24 was 4.8 (SD, 8.1) in cohort 1 and 7.5 (SD, 7.6) in cohort 2, which continued to stabilize to Week 48, with improvement seen from Week 72 (Table 4). At Week 48, one (20.0%; 1/5) and nine (56.3%; 9/17) patients had an improvement in SDMT score of ≥ 8 points from baseline in cohorts 1 and 2, respectively; at Week 96, the corresponding numbers of patients were three (100.0%; 3/3) and 10 (62.5%; 10/17).

## Discussion

The objective of the OPERETTA I study was to identify the appropriate OCR dosing regimen for pediatric patients with RRMS for further evaluation of safety and efficacy in the phase 3 OPERETTA II study (NCT05123703), which is currently ongoing. Based on the data obtained in this study, the selected dosing regimen was 300 mg OCR IV for patients with a BW < 35 kg and 600 mg OCR IV for patients with a BW ≥ 35 kg, administered every 24 weeks. This dosing regimen is expected to provide similar OCR exposure to pediatric patients as observed in adult patients with MS at the approved dose of 600 mg IV.

OCR treatment with the identified dose led to rapid and near-complete CD19^+^ B-cell depletion in blood, which was sustained throughout the study and was similar to the data collected from the OPERA I/II trials in adult patients with RRMS receiving a dose of OCR 600 mg IV [19].

Overall, OCR was well tolerated in pediatric patients. The safety profile of OCR in children and adolescents in this study in both treatment arms was similar to the known safety profile of OCR in adults [19]. All 23 enrolled patients completed the 24-week DEP and continued in the OOE; as of the CCOD, only one patient had withdrawn from the study (withdrawal of consent/assent by participant). No patient developed treatment-emergent ADAs.

While this study was not powered to demonstrate efficacy, the results of exploratory clinical and imaging endpoints observed in both treatment arms during the DEP and OOE period (up to 264 weeks) show that OCR leads to good control of disease activity. No relapses occurred in either of the cohorts after baseline, including during the DEP and OOE period. Moreover, EDSS scores numerically decreased from baseline to Weeks 48 and 96 in both treatment cohorts, and NEDA was observed in the majority of patients at Weeks 48 and 96, although it is important to note the small sample size and very limited range of EDSS scores.

In our study, no new T1 Gd-enhancing lesions were observed at Week 12 after first dose of OCR in either cohort. The formation of N/E T2 lesions declined over time, with no N/E T2 lesions observed after Week 24 in cohort 1. In cohort 2, a continuous decline in N/E lesion count was observed from Week 12, with no new lesions observed after Week 48 among the 17 patients continuing in the OOE. Overall, the number of N/E T2 lesions decreased over time in both cohorts, with a complete stop in new lesion formation after Week 12 for cohort 1 and after Week 48 for cohort 2, signifying MS condition improvement.

Cognition is recognized to be an important feature of MS, and research has shown that patients with POMS experience more rapid reduction in information processing efficiency over time and are more likely to experience cognitive impairment than patients with AOMS, independent of age or disease duration [4]. In patients with POMS, the SDMT is a widely used, reliable, and effective screening tool for cognitive impairment [20]. Indeed, recent literature related to the validity of the SDMT has further supported its use as the best candidate for a cognition outcome measure [21, 22]. In the OPERETTA I study, we observed similar SDMT scores to those observed in the pediatric MS population in a study evaluating SDMT as a tool for identifying POMS patients at risk for cognitive impairment [21]. While clinicians frequently denote a decline of 4 points on the SDMT as meaningful in the context of learning and cognitive development with age, higher cutoffs may be needed to demonstrate a statistically significant decline at an individual (vs group) level [23]. Thus, due to the small sample size in the OPERETTA I study, an 8-point cutoff may be considered adequate. By Week 96, three (100.0%; 3/3) patients in cohort 1 and 10 (62.5%; 10/17) patients in cohort 2 had an improvement in SDMT score of ≥ 8 points from baseline; however, the effects of cognitive maturation with increased age or learning effects with repeated testing have not been corrected for.

Given the nature of this phase 2 PK/PD study, all efficacy results need to be interpreted in the context of the limitations of the small sample size and the exploratory nature of the efficacy objectives. The favorable outcomes observed in OPERETTA I for all exploratory outcome measures suggest that OCR could provide a highly effective and well-tolerated treatment option for pediatric patients with MS. If confirmed in the ongoing phase 3 OPERETTA II study, these results demonstrate that OCR would be of high therapeutic benefit to patients with POMS, as this patient population shows a more active disease course compared with AOMS, with an annualized relapse rate of 1.13 vs 0.40; *p* < 0.001 [24], higher lesion load at disease onset, and a higher rate of new MRI lesion formation [25]. A recent retrospective cohort study involving > 3800 pediatric patients with RRMS also highlighted the benefit of treatment with high-efficacy DMTs vs moderately effective therapies [26]. In this study, highly effective therapy dampened disease activity, with a 54% reduction in first relapse risk and with optimal effect within the first 2 years [26].

Regarding the OPERETTA I study conduct, the long study duration reflects the challenges of conducting clinical trials in POMS, given the rarity of MS onset prior to 18 years of age and the rarity of patients with POMS presenting with a BW under 40 kg and prior to puberty. In addition, challenges with enrollment may reflect the need for greater engagement with parent and family groups; the involvement of parent groups as active participants in trial design may have a positive effect on study participation.

## Conclusions

The results of this study led to the selection of the following dosing regimen for the OPERETTA II study: 300 mg OCR IV for patients with a BW < 35 kg and 600 mg OCR IV for patients with a BW ≥ 35 kg, administered every 24 weeks. If the favorable tolerability, safety, and efficacy profiles are confirmed in the ongoing phase 3 trial, OCR may be a promising new treatment option for pediatric patients with RRMS.

## Supplementary Information

Below is the link to the electronic supplementary material.

Supplementary file1 (DOCX 21 KB)
