# Supplementary material for: Ocrelizumab dose selection for treatment of pediatric relapsing–remitting multiple sclerosis: results of the OPERETTA I study
Source: J Neurol. 2025 Jan 15;272(2):137. doi: 10.1007/s00415-024-12879-z (PMC11735518; doi:10.1007/s00415-024-12879-z)
Supplement: Supplementary file 1 — Supplementary file1 (DOCX 21 KB) [file 415_2024_12879_MOESM1_ESM.docx]

# **Supplementary Information**

## **Methods**

## **Criteria for retreatment with ocrelizumab**

Prior to retreatment, the following conditions must be met:

- Absence of life-threatening (grade 4) infusion-related event that occurred during a previous ocrelizumab infusion
- Absence of severe allergic or anaphylactic reaction to an ocrelizumab infusion
- Absence of active infection, regardless of the grade (including active tuberculosis infection, either new onset or reactivation), and treatment with any anti-infective medications has been completed
- Absence of any significant or uncontrolled medical condition or treatment-emergent, clinically significant laboratory abnormality
- Absolute neutrophil count ≥1.5 × 103 μ/L
- Immunoglobulin G (IgG) level:
  - For children and adolescents: IgG ≥4.6 g/L
  - For patients ≥18 years of age, the adult reference applies: IgG ≥3.3 g/L
- No initiation of protocol-prohibited medications
- Body weight ≥25 kg
- Absence of ongoing pregnancy or positive pregnancy test or breastfeeding (for female patients)

If any of these conditions are not met prior to redosing, further administration of ocrelizumab will be suspended until events are resolved and criteria are met, or ocrelizumab will be held off indefinitely.

## **Key inclusion criteria**

### **Inclusion criteria:**

- Body weight ≥25 kg
- Children and adolescents must have received all childhood required vaccinations
- Female participants of childbearing potential must agree to either remain completely abstinent or to use reliable means of contraception
- Diagnosis of relapsing-remitting multiple sclerosis
- Expanded Disability Status Scale score at screening: 0–5.5, inclusive
- Neurologic stability for ≥30 days prior to screening, and between screening and baseline
- Participants naive to prior disease-modifying therapy (DMT)
- Participants who have had at least 6 contiguous months of DMT within the past 1 year must have evidence of disease activity occurring after the full 6-month course of treatment, that is, at least one relapse or ≥1 gadolinium-enhancing lesion on a T1-weighted brain MRI

## **Supplementary Table S1** Number of patients < or ≥ LLN CD19 Ab cell count

|  | **OCR 300 mg (n=6)** | **OCR 600 mg (n=17)** |
| --- | --- | --- |
| **Week 2, n (%)** | **5** | **15** |
| <LLN  ≥LLN | 5 (100.0)  0 (0.0) | 15 (100.0)  0 (0.0) |
| **Week 4, n (%)** | **6** | **15** |
| <LLN  ≥LLN | 6 (100.0)  0 (0.0) | 15 (100.0)  0 (0.0) |
| **Week 8, n (%)** | **6** | **16** |
| <LLN  ≥LLN | 6 (100.0)  0 (0.0) | 16 (100.0)  0 (0.0) |
| **Week 12, n (%)** | **6** | **16** |
| <LLN  ≥LLN | 6 (100.0)  0 (0.0) | 16 (100.0)  0 (0.0) |
| **Delayed dosing – Dose 1 infusion 2, n (%)** | **0** | **1** |
| <LLN  ≥LLN | 0 (0.0)  0 (0.0) | 1 (100.0)  0 (0.0) |
| **Week 16, n (%)** | **6** | **16** |
| <LLN  ≥LLN | 6 (100.0)  0 (0.0) | 16 (100.0)  0 (0.0) |
| **Week 22, n (%)** | **0** | **1** |
| <LLN  ≥LLN | 0 (0.0)  0 (0.0) | 0 (0.0)  1 (100.0) |
| **Week 24, n (%)** | **6** | **17** |
| <LLN  ≥LLN | 5 (83.3)  1 (16.7) | 16 (94.1)  1 (5.9) |
| **Week 36 OOE, n (%)** | **4** | **13** |
| <LLN  ≥LLN | 4 (100.0)  0 (0.0) | 13 (100.0)  0 (0.0) |
| **Week 46 OOE, n (%)** | **5** | **17** |
| <LLN  ≥LLN | 4 (80.0)  1 (20.0) | 17 (100.0)  0 (0.0) |
| **Week 60 OOE, n (%)** | **3** | **14** |
| <LLN  ≥LLN | 3 (100.0)  0 (0.0) | 14 (100.0)  0 (0.0) |
| **Week 70 OOE, n (%)** | **3** | **17** |
| <LLN  ≥LLN | 3 (100.0)  0 (0.0) | 17 (100.0)  0 (0.0) |
| **Week 84 OOE, n (%)** | **3** | **12** |
| <LLN  ≥LLN | 3 (100.0)  0 (0.0) | 12 (100.0)  0 (0.0) |
| **Week 94 OOE, n (%)** | **4** | **15** |
| <LLN  ≥LLN | 3 (75.0)  1 (25.0) | 15 (100.0)  0 (0.0) |
| **Week 96 OOE, n (%)** | **0** | **1** |
| <LLN  ≥LLN | 0 (0.0)  0 (0.0) | 1 (100.0)  0 (0.0) |
| **Week 108 OOE, n (%)** | **2** | **15** |
| <LLN  ≥LLN | 2 (100.0)  0 (0.0) | 15 (100.0)  0 (0.0) |

Age-appropriate LLNs: age 9–13 years, LLN = 170 cells/µL; age 14–17 years,
LLN = 140 cells/µL [1]; and age ≥18 years, LLN = 80 cells/µL [2].

Repleted is defined as CD19 ≥ LLN or baseline, whichever is lower.

Abbreviations: LLN, lower limit of normal; OCR, ocrelizumab; OOE, optional ocrelizumab extension period.

**Supplementary references**

1. Garcia-Prat M, Álvarez-Sierra D, Aguiló-Cucurull A, Salgado-Perandrés S, Briongos-Sebastian S, Franco-Jarava C, Martin-Nalda A, Colobran R, Montserrat I, Hernández-González M, Pujol-Borrell R, Soler-Palacin P, Martínez-Gallo M (2019) Extended immunophenotyping reference values in a healthy pediatric population. Cytometry B Clin Cytom 96:223–233. doi:10.1002/cyto.b.21728.
2. Gibiansky E, Petry C, Mercier F, Günther A, Herman A, Kappos L, Hauser S, Yamamoto Y, Wang Q, Model F, Kletzl H (2021) Ocrelizumab in relapsing and primary progressive multiple sclerosis: pharmacokinetic and pharmacodynamic analyses of OPERA I, OPERA II and ORATORIO. Br J Clin Pharmacol 87:2511–2520. doi: 10.1111/bcp.14658.
